# A small secreted protein from *Zymoseptoria tritici* interacts with a wheat E3 ubiquitin ligase to promote disease

**DOI:** 10.1093/jxb/eraa489

**Published:** 2020-10-23

**Authors:** Sujit Jung Karki, Aisling Reilly, Binbin Zhou, Maurizio Mascarello, James Burke, Fiona Doohan, Dimitar Douchkov, Patrick Schweizer, Angela Feechan

**Affiliations:** 1 School of Agriculture & Food Science and UCD Earth Institute, University College Dublin, Belfield, Dublin 4, Ireland; 2 School of Biology and Environmental Science and UCD Earth Institute, University College Dublin, Belfield, Dublin 4, Ireland; 3 Ecology, Evolution and Biodiversity Conservation, Charles Deberiotstraat 8 32, 3000 Leuven, Belgium; 4 Institute of Plant Genetics and Crop Plant Research (IPK), Cytogenetics, Gatersleben, Germany; 5 University of Birmingham, UK

**Keywords:** Disease, effector, E3 ubiquitin ligase, fungal pathogen, wheat, *Zymoseptoria tritici*

## Abstract

Septoria tritici blotch (STB), caused by the ascomycete fungus *Zymoseptoria tritici*, is a major threat to wheat production worldwide. The *Z. tritici* genome encodes many small secreted proteins (ZtSSPs) that are likely to play a key role in the successful colonization of host tissues. However, few of these ZtSSPs have been functionally characterized for their role during infection. In this study, we identified and characterized a small, conserved cysteine-rich secreted effector from *Z. tritici* which has homologues in other plant pathogens in the Dothideomycetes*. ZtSSP2* was expressed throughout *Z. tritici* infection in wheat, with the highest levels observed early during infection. A yeast two-hybrid assay revealed an interaction between ZtSSP2 and wheat E3 ubiquitin ligase (TaE3UBQ) in yeast, and this was further confirmed *in planta* using bimolecular fluorescence complementation and co-immunoprecipitation. Down-regulation of this wheat E3 ligase using virus-induced gene silencing increased the susceptibility of wheat to STB. Together, these results suggest that TaE3UBQ is likely to play a role in plant immunity to defend against *Z. tritici.*

## Teaser

A conserved secreted effector from the fungal pathogen *Zymoseptoria tritici* (ZtSSP2) promotes disease in wheat through an interaction with an E3 ubiquitin ligase.

## Introduction

Plant innate immunity includes the recognition of broadly conserved pathogen- associated molecular patterns (PAMPs), for example fungal chitin, by plant pattern recognition receptors. This recognition initiates PAMP-triggered immunity (PTI) to mount a primary defence (Jones and Dangl, 2006). Pathogen effector proteins have evolved to bypass the initial defence response (PTI), resulting in a scenario termed effector triggered susceptibility. These proteins are typically small, cysteine-rich secreted proteins, and are known to manipulate host physiology and interfere with plant immunity (Dou and Zhou, 2012). In return, plants possess resistance genes which, upon recognition of pathogen effectors, activate effector triggered immunity (Jones and Dangl, 2006; Deslandes and Rivas, 2012).

Septoria tritici blotch (STB) caused by *Zymoseptoria tritici* is one of the most prevalent and economically devastating diseases in wheat-growing areas worldwide (Eyal *et al.*, 1985; Fones and Gurr, 2015). As with other fungal pathogens, *Z. tritici* is known to produce a series of small secreted effector proteins (SSPs) throughout its colonization of wheat (Morais do Amaral *et al.*, 2012; Mirzadi Gohari *et al.*, 2015; Rudd *et al.*, 2015). Only a handful of *Z. tritici* effectors have been characterized for their role in pathogenesis: Mg3LysM and Mg1LysM are lysin motif-containing effectors that play an important role during the initial symptomless period of *Z. tritici* infection (Marshall *et al.*, 2011). Mg3LysM competes with host chitin receptors by binding fungal chitin fragments (Lee *et al.*, 2014). Another *Z. tritici* effector, MgNLP, belongs to the Necrosis and Ethylene-Inducing Peptide 1 (NEP1)-like (NLP) family of proteins. It can induce cell death in Arabidopsis, but not in wheat (Motteram *et al.*, 2009). In addition, Z*. tritici* secretes two necrosis-inducing protein effectors (ZtNIP1 and ZtNIP2) that induce cell death and chlorosis in some wheat cultivars (Ben M’ Barek *et al*., 2015).

Recently, several *Z. tritici* candidate effectors were found to induce a cell death phenotype when expressed in the non-host plant *Nicotiana benthamiana* (Kettles *et al.*, 2017). One of these candidate effectors was subsequently identified as a novel fungal PAMP, termed Cell Death-Inducing 1 (ZtCDI1) (Franco-Orozco *et al.*, 2017). Similarly, another candidate (Zt-6) was characterized as a secreted RNase which possesses cytotoxic activity against other microbes as well as plants (Kettles *et al.*, 2018). Additionally, avirulence effectors, namely Avrstb6 (Zhong *et al.*, 2017, Kema *et al.*, 2018) and Avr3D1 (Meile *et al.*, 2018), are known to be recognized by specific wheat cultivars. Recently, *Z. tritici* SSPs were found to interact with wheat septoria-responsive taxonomically restricted genes, namely *TaSRTRG6* and *TaSRTRG7*, implicated in STB disease (Brennan *et al.*, 2020). However, the function of these taxonomically restricted genes is unknown. While some *Z. tritici* candidate effectors have been identified, the molecular targets in wheat and their role during infection remain largely unknown. Therefore, there is a need to identify the wheat host proteins targeted by these *Z. tritici* effectors.

In this study, we selected a conserved effector candidate (ZtSSP2) similarly expressed in three different *Z. tritici* isolates. ZtSSP2 is a small secreted protein (22 kDa) with 10 cysteine residues that appears to be conserved within *Z. tritici* isolates and other Dothideomycete fungi. Furthermore, we showed that ZtSSP2 physically interacts with wheat E3 ubiquitin ligase (TaE3UBQ) and that virus-induced gene silencing (VIGS) of *TaE3UBQ* resulted in increased *Z. tritici* susceptibility. This study provides insights in to how a *Z. tritici* effector is likely to target a host ubiquitin system to aid successful colonization.

## Materials and methods

### Plant material, fungal strains, and growth conditions

*Nicotiana benthamiana* and wheat (*Triticum aestivum*) cvs Remus, Kanzler, and Longbow were used in this study. The cvs Remus and Kanzler are moderately susceptible to *Z. tritici* (Brady, 1983; Váry *et al.*, 2015) while cv. Longbow is susceptible to *Z. tritici* (Brading *et al.*, 2002). *Nicotiana benthamiana* plants were grown in growth chambers with 16 h of light at 22 °C/8 h of dark at 18 °C with 7500–8200 lux, relative humidity 70±5% throughout the experiments and 4- to 6-week-old *N. benthamiana* plants were used for localization studies.

Wheat seeds (cvs Remus and Longbow) were surface-sterilized and incubated for 3 d at 4 °C for seed stratification and then incubated for 4 d at room temperature without illumination to allow germination. Germinated seeds were transferred into plastic pots containing John Innes Compost No. 2 (Westland Horticulture, UK) and grown in a growth chamber at a 16 h day/8 h night photoperiod at 13 000 lux, relative humidity 80±5% at 19 °C/12 °C. For biolistic studies, wheat cv. Kanzler seedlings were grown in a growth chamber at a 16 h day/8 h night photoperiod at 15 000 lux, relative humidity 60±5% at 20 °C in pots containing IPK soil substrate.

The *Z. tritici* isolate IPO323 (Kema and van Silfhout, 1997) and Irish isolates 560.11 (Lynch *et al.*, 2016) and 553.11 were used to infect the susceptible wheat cvs Remus and Longbow. Both 560.11 and 553.11 were isolated from the wheat cv. Alchemy in 2011 from South Wexford, Ireland (S. Kildea, personal communication). Prior to use, isolates were cultured on potato dextrose agar (PDA) and grown at 20 °C for ~5–7 d. Fourteen-day-old wheat seedlings were used for inoculation. For the disease assay, we used a 10^6^ cfu ml^–1^ spore suspension as inoculum (Shetty *et al.*, 2003; Fones *et al.*, 2017; Zhong *et al.*, 2017) to assess pycnidia and necrosis coverage following VIGS of *TaE3UBQ*. Fungal spores from PDA cultures were harvested and spore concentration was adjusted to 1×10^6^ ml^−1^ in water containing 0.02% Tween-20. Spore suspensions (5 ml per plant) were sprayed to runoff (5 ml) per wheat plant using hand-held spray bottles. Control plants were sprayed with 5 ml of 0.02% Tween-20 solution. Inoculated plants were covered with polythene bags to ensure high humidity, and removed after 72 h.

### Effector candidate selection

The publicly available secretome dataset from Morais do Amaral *et al.*, 2012 which is based on the IPO323 reference genome (Goodwin *et al.*, 2011), was mined to identify *ZtSSP* genes. A total of 262 candidate genes with EST support were screened based on small size (50–315 amino acids), resulting in 102 SSPs. These were sorted based on the number of cysteine residues, which resulted in 90 SSPs with multiple cysteines (see Supplementary Fig. S1 at *JXB* online). The amino acid sequence was then used to predict effector properties and any apoplastic localization using EffectorP & ApoplasticP (Supplementary Table S1) (Sperschneider *et al*., 2016, 2018). These were analysed using NCBI CDD (Conserved Domain Database) to update the prediction of any conserved domains. Candidate effector proteins were analysed using BLASTP (cut-off value ≥50% identity and e-value ≤0.01) to search for homologues in other plant pathogenic fungal species (Supplementary Table S2). Finally, those that were unannotated (Supplementary Table S1) and with a potential homologue in other plant pathogenic fungi were selected. Of these, 17 non-annotated *ZtSSP* genes were analysed for expression among three *Z. tritici* isolates (IPO323, 553.11, and 560.11) at 7 days post-infection (dpi) (Supplementary Table S3). *Mycgr3G105265* (*ZtSSP2*) was selected for further study based on these criteria and the similar expression levels across all three isolates.

### Transcriptomics analysis

Leaves (cv. Longbow) infected with the *Z. tritici* isolates IPO323, 553.11, and 560.11 from four independent replicates were collected at 7 dpi. RNA from each sample was extracted at room temperature using the RNeasy Plant Mini Kit (QIAGEN), purified from DNA contamination using DNase I (Sigma Aldrich), and stored at 20 °C. RNA quality control was assessed by Agilent 2100 bioanalyzer. RNA was extracted from two leaves from each of two seedlings infected with each isolate over four independent experiments (*n*=16), pooled, and sent for RNA sequencing on an Illumina HiSeq 2000 (paired-end 100 bp reads) at the 250 Bejing Genome Institute (BGI) (Hong Kong).

Expression analysis was performed by General Bioinformatics (Reading, UK). The reference genome for *Z. tritici* was from the MG2 assembly from ENSEMBL Fungi release. The reference genome for *T. aestivum* was from the IWGSC1+popseq assembly from ENSEMBL Plants release. The quality control of raw reads was assessed with FastQC v0.11.5 (Andrews, 2010). Reads were trimmed to remove contaminating adapter sequences and poor-quality bases at the beginning of the reads using Trimommatic (Bolger *et al.*, 2014). Clean reads generated were 185 725 832 for IPO323 isolate-infected leaf samples, 187 073 058 for 553.11 isolate-infected leaf samples, and 183 036 662 for the 560.11 isolate-infected leaf sample. Unfiltered reads were aligned to the *Z. tritici* genome using Tophat v2.1.1 aligner (Kim *et al.*, 2013). BAM files for reads mapped to the *Z. tritici* genome were converted to SAM files and sorted for further analysis using Samtools v 1.3 (Li *et al.*, 2009). Then, reads were counted using the htseq-count script of the HTSeq v 0.6.0 (Anders *et al.*, 2015). Fragments per kilobase of transcript per million mapped reads (FPKM) values for each sample were calculated using the Cufflink package (Trapnell *et al.*, 2010). Genes with a total read count <1 were filtered out. Counts were normalized using TMM (Robinson *et al.*, 2010), and the common dispersion BCV (square root dispersion) was set at 0.4. Pairwise comparisons between datasets were made using the exact test (Robinson *et al.*, 2008). Filtered reads were aligned to the *T. aestivum* genome using Tophat v2.1.1. The BAM file containing the unmapped reads was converted back to FastQ format using the bam2fastx utility of Tophat (Kim *et al.*, 2013). The alignment to the *Z. tritici* genome and subsequent analysis were performed as described for the unfiltered reads.

Expression of *TaE3UBQ* homeologues was determined using expVIP (expression Visualization and Integration Platform) (Borrill *et al.*, 2016).

### Amplification and cloning of *ZtSSP*2

*ZtSSP2* was amplified from wheat (cv. Remus) infected with *Z, tritici* isolate 560.11 cDNA with and without the signal peptide using Phusion High Fidelity Polymerase (New England Biolabs) and primers flanked with Gateway adapter sequence (Supplementary Table S4). AttB-flanked PCR products were purified using the QIA quick PCR Purification Kit (Qiagen), cloned into pDONR207 (Invitrogen) using BP clonase II enzyme mix (Thermo Fisher Scientific), and subsequently cloned into the binary vector pEARLYGATE 101 (pEG101) (Earley *et al.*, 2006) using LR clonase II enzyme. Entry clones were sequence verified by sequencing (Macrogen Europe) before LR reaction. All other destination vectors are described separately.

### Validation of protein secretion using a yeast sucrose secretion system

A Gateway-compatible vector (pGADT7) for yeast secretion assay and *suc2* yeast mutant (strain SEY6210) was utilized (Brennan *et al.*, 2020). Briefly, the invertase (*SUC2*) gene with and without signal peptide was amplified from the yeast strain BY4741 with a linker (Kex2 site) added between the Gateway reading frame and the *SUC2* gene. This construct was ligated into the pGADT7 vector and verified by sequencing. Candidate *ZtSSP2* and *ΔSP-ZtSSP2* were cloned into the yeast secretion vector in-frame with the N-terminus of the *SUC* gene and transformed into the *suc2* yeast mutant. Transformants were PCR validated and selected on a synthetic dropout medium (minus Trp and Leu) with sucrose as a sole carbon source. Yeast spotting was performed with dilutions of 10^–1^, 10^–2^, and 10^–3^, respectively. The experiment was repeated three times independently with three replicates per experiment.

### *In silico* analysis

BLASTp search was performed using the NCBI (National Centre for Biotechnology Information) BLAST service (http://blast.ncbi.nlm.nih.gov/Blast.cgi) and uniprot blast (http://www.uniprot.org/blast/). Sequences of the 20 closest homologues of ZtSSP2 (Supplementary Table S5) were aligned using ClustalW, and a phylogenetic tree was constructed using the maximum likelihood (ML) method with 1000 bootstrap replicates in Mega7 (Kumar *et al.*, 2016). Prediction of conserved domains, transmembrane domains, and protein structure was performed with NCBI CDD, TMHMM Server v2 (http://www.cbs.dtu.dk/services/TMHMM/), and MemBrain 3.1, respectively.

### RNA extraction and quantitative RT-PCR

A 100 mg aliquot of infected leaves (cv. Remus) per sample was collected at different days post-infection, frozen in liquid nitrogen, and stored at –80 °C. Total RNA was extracted from *Z. tritici*-infected wheat leaves using the RNeasy Mini Kit (Qiagen) following the manufacturer’s instructions. RNA was then subjected to on-column DNase treatment (Sigma). Quantification of total RNA was carried out using a Nanodrop ND-1000 spectrophotometer. Reverse transcription of 1–2 µg of RNA for cDNA synthesis was carried out using the Omniscript RT Kit (Qiagen).

Real-time quantitative PCR (qRT-PCR) was carried out in 12.5 µl reactions including 1.25 μl of a 1:5 (v/v) dilution of cDNA, 0.2 μM of primers, and 1×SYBR Premix Ex Taq (Tli RNase H plus, RR420A; Takara). PCR conditions were as follows: 1 cycle of 1 min at 95 °C; 40 cycles of 5 s at 95 °C and 20 s at 60 °C; and a final cycle of 1 min at 95 °C, 30 s at 55 °C, and 30 s at 95 °C for the dissociation curve. For *ZtSSP2* expression, RNA was extracted from the third leaf of wheat seedlings and from three individual leaves from different seedlings per time point per replicate. Three independent experiments were performed. qPCR was performed using the QuantStudio 7 Flex Real-Time PCR system (Applied Biosystems) and the relative gene expression was calculated as 2^−(Ct target gene–Ct housekeeping gene)^ as previously described (Livak and Schmittgen, 2001). The *Z. tritici* tubulin gene was used as housekeeping gene control for the ZtSSP2 time course. For VIGS, Ct housekeeping gene=geometric mean (Ct *Tacdc48*:Ct *Taelf4E*) of two wheat reference genes: cell division control protein 48 (*TaCDC48*) and Eukaryotic Initiation factor 4E (*TaeIF4E)* (Lee *et al.*, 2014).

### Single-cell death assay in wheat

The cell death assay in wheat was performed as previously described (Pliego *et al.*, 2013). Briefly, seven leaves of 7-day-old wheat (~8 cm in length) cv. Kanzler were co-bombarded (PDS-1000/He System, Bio-Rad) with 7 µg of pEG101:(ΔSP) *ZtSSP2* (overexpression), 7 µg of pUbiGUS (β-glucuronidase reporter for transformation efficiency), and 7 µg of the *B-Peru/C1*-expression plasmid pBC17 (Schweizer *et al.*, 2000) for induction of anthocyanin production (as a marker for live cells). Four days post-bombardment, cells accumulating anthocyanin were counted and leaves were then stained with 5-bromo-4-chloro-3-indolyl glucuronide (X-gluc) solution overnight at 37 °C. Leaves were destained with trichloroacetic acid (Douchkov *et al.*, 2005). The number of cells with visible GUS stain was counted and the relative number of anthocyanin-producing cells was calculated as the ratio of anthocyanin-accumulated cells to the number of GUS-expressing cells. The experiment was repeated four times independently and seven leaves were counted per replicate.

### Yeast two-hybrid analysis

The cDNA library was comprised of leaf three of wheat seedlings cvs Stigg and Longbow which were infected with a mixture of *Z. tritici* isolates (IPO323, 560.11, and Cork cordiale 4) (Kema *et al.*, 1997; Lynch *et al.*, 2016; Brennan *et al.*, 2020), collected at various time points (1, 2, 4, 6, 8, 10, and 12 dpi) then pooled together for RNA extraction. Briefly, the cDNA of (ΔSP) *ZtSSP2* was cloned into the pB27 vector as an N-LexA-bait-C fusion to LexA. The construct encoding ZtSSP2 was used as bait to screen the cDNA library of wheat leaves inoculated with *Z. tritici*.

The initial yeast two-hybrid screening was performed by Hybrigenics Services, S.A.S. (http://www.hybrigenics-services.com). A total of 76.4 million clones were screened and 73 positive clones were processed following selection on selective medium lacking Trp, Leu, and His supplemented with 0.5 mM 3-amino-1,2,4-triazole (3AT). The prey fragments from positive clones were amplified and sequenced. The putative high confidence interactors are listed in Supplementary Table S6. These sequences were used to identify corresponding proteins in the NCBI GenBank database including TaE3UBQ. For analysis of a specific interaction, the coding sequence of *TaE3UBQ*, *TaE3UBQ*_126–219_, the barley homologue of E3UBQ (HvUBQ), (ΔSP) ZtSSP2, and the *Ramularia collo-cygni* homologue of ZtSSP2 (RcSSP2) was cloned into the vector pDONR207 using Gateway cloning technology. They were then recombined into bait and prey vectors derived from pGADT7 and pGBKT7 plasmids (Clontech, USA). Analysis of protein–protein interactions was performed using the Gal4 two-hybrid assay as described in Perochon *et al.* (2010). As a negative control to ensure specific interactions with ZtSSP2, another small secreted effector candidate (ΔSP) Zt-10 was used (Kettles *et al.*, 2017), while the positive control included TaSSP6 and Zt-06 (Zhou *et al.*, 2020).

### *Agrobacterium-*mediated transient expression

*Agrobacterium tumefaciens* strain GV3101 was transformed by electroporation with effector constructs and grown for 48 h at 28 °C at 220 rpm in LB medium with the antibiotics gentamicin (25 mg ml^–1^) and kanamycin (50 mg ml^–1^). Transformed cells were harvested by centrifugation and suspended in infiltration buffer (10 mM MgCl_2,_ 10 mM MES, pH 5.6, and 150 µM acetosyringone) at an absorbance at 600 nm (OD_600_) of 0.5. The bacterial suspension was left at room temperature for 2 h before infiltration into 4- to 6-week-old *N. benthamiana* on the abaxial side of the leaves using a 1 ml needleless syringe.

### *In planta* validation of protein–protein interaction

For *in planta* analysis of the interaction between (ΔSP) ZtSSP2 and TaE3UBQ, the coding sequences were cloned in the Gateway vector pDONR207 (Invitrogen, USA) and subsequently cloned into the bimolecular fluorescence complementation (BiFC) vectors pDEST-GW VYCE, pDEST-VYCE GW, pDEST-GW VYNE, and pDEST-VYNE GW (Gehl *et al.*, 2009). This resulted in constructs where proteins were fused at either the N- or C-terminus to the yellow fluorescent protein C-terminal (YFP^C^) or N-terminal fragment (YFP^N^). For localization of TaE3UBQ, LR reaction was performed with pGWB406 (Nakagawa *et al.*, 2007). Vectors were transformed into *A. tumefaciens* strain GV3101 by electroporation. Transformants containing the plasmids were selected on LB agar plates containing 10 µg ml^−1^ rifampicin, 20 µg ml^−1^ gentamicin, and 50 µg ml^−1^ kanamycin. A mix of *Agrobacterium* transformants was prepared: OD_600_=0.5, 0.5, and 0.1 of YFP^C^ construct, YFP^N^, and P19 silencing construct, respectively. This mix was syringe-infiltrated into leaf epidermal cells of 3- to 4-week-old *N. benthamiana* by making a small injury to the leaf and pressure infiltrating. For TaE3UBQ localization, MG132 (100 µM) was infiltrated into leaves for 6 h before analysis to prevent protein degradation. Images were analysed using a confocal laser scanning microscope (Olympus fluoview FV1000). Green fluorescent protein (GFP) and YFP excitation was performed at 515 nm and emission detected in the 530–630 nm range. These experiments were repeated at least twice independently, and each experiment included three leaves, each from an individual plant.

### Co-immunoprecipitation (Co-IP) assay

The protein construct was transiently overexpressed in *N*. *benthamiana* leaves using agro-infiltration. Leaf samples were collected at 48 h post-infiltration, and TaE3UBQ and GFP samples were infiltrated with MG132 (100 µM) 6 h before collection. Proteins were extracted using GTEN buffer (25 mM Tris–HCl pH 7.5, 150 mM NaCl, 1 mM EDTA, 10% glycerol 0.1% Tween-20) with 2% (w/v) polyvinylpolypyrrolidone (PVPP), 10 mM DTT, and a protease inhibitor cocktail (Sigma). Samples were incubated for 15 min in lysis buffer (4 °C). Lysate was centrifuged at 10 000 *g* for 10 min and 250 µl of supernatant was subjected to Co-IP with GFP-Trap^®^-M magnetic beads (Chromotek, Germany) for affinity binding of GFP-fused proteins at 4 °C for 4 h. The beads were washed three times with 500 µl of extraction buffer. Protein bound to magnetic beads was boiled for 10 min for elution. Eluted proteins and crude proteins (input) were detected by western blotting. Immunoblotting of the proteins on the PVDF membrane were detected using the corresponding anti-HA (1:1000; Roche) and anti-GFP (1:5000; Invitrogen) antibodies.

### BSMV-mediated gene silencing

The *Barley stripe mosaic virus* (BSMV)-derived VIGS vectors used in this study consisted of the wild-type BSMV ND18 α, β, γ tripartite genome (Holzberg *et al.*, 2002; Scofield *et al.*, 2005). A BSMV γ vector construct containing a 185 bp fragment of the barley phytoene desaturase gene (BSMV:PDS) was used as positive control for VIGS, as previously described (Scofield *et al.*, 2005) (Supplementary Fig. S2). Two independent, non-overlapping gene constructs (UBQV1 and UBQV2) were used for gene silencing (Supplementary Fig. S3). Both gene fragments were PCR-amplified and chosen to target *TaE3UBQ-1D* and its homoalleles (*TaE3UBQ-1A* and *TaE3UBQ-1B*). The specificity and silencing efficiency were evaluated by BLASTn and using the SGN VIGS tool with *T. aestivum IWGSC2* as a database (Fernandez-Pozo *et al.*, 2015). PCR-amplified fragments were ligated in the antisense orientation into *Not*I/*Pac*I-digested BSMV γ vector pSL038-1 (Scofield *et al.*, 2005). Construct authenticity was verified by sequencing.

Vectors containing the BSMV α, γ genomes and the γ genome vectors containing either BSMV:UBQV1, BSMV:UBQV2, or BSMV:PDS were linearized with *Mlu*I. The BSMV β genome was linearized with *Spe*I. Capped *in vitro* transcripts were prepared from the linearized plasmids using the mMessage mMachine T7 *in vitro* transcription kit (AM1344, Ambion) following the manufacturer’s protocol. RNA quantity and quality were evaluated using the ND-1000 spectrophotometer (NanoDrop, Thermo Fisher Scientific, USA) measurement. Capped BSMV transcripts (1:1:1) with 1× FES buffer were rub-inoculated onto the second leaf of the two-leaf stage of the wheat cv. Longbow. BSMV:PDS was used as positive control, whereas BSMV γ and 1× FES buffer were used as negative control. Fourteen days after virus inoculation, the third and fourth leaves of virus-inoculated wheat seedlings (12 plants per treatment per trial) were infected with *Z. tritici* (560.11). The third leaves were collected at 10 dpi and RNA extracted individually from each of the leaves per treatment. The fourth leaves were used for STB disease symptom phenotyping at 21 dpi (10 leaves per treatment, three independent trials). Seven leaves per treatment per replicate were submerged in 10 ml of deionized water for 1 h and vortexed to collect spores, and 10 µl was counted using a haemocytometer.

### Statistical analysis

Statistical analysis of the data was carried out using the R statistical software (R Core Team, 2016). All data from the studies were checked for normal distribution and, when necessary, variances were stabilized using Box–Cox transformation. A generalized linear model was used to test the data, and significant differences were determined using the Tukey test at *P*<0.05. For analysis of VIGS data (phenotyping and spore counts), data were fitted to a generalized linear mixed model with binomial distribution to account for overdispersion and zero inflation. Significance of differences between treatments was assessed using the Tukey’s HSD.

## Results

### ZtSSP2 is a conserved small secreted protein candidate

The publicly available secretome dataset from Morais do Amaral *et al.* (2012) was mined to identify *Z. tritici* small secreted proteins (ZtSSPs). A total of 262 candidate genes with EST support were screened based on small size (50–315 amino acids), resulting in 102 ZtSSPs. These proteins were then sorted based on the number of cysteine residues (≥1), which resulted in 90 ZtSSPs (Supplementary Fig. S1). The amino acid sequence was then used to predict effector properties and any apoplastic localization using EffectorP & ApoplasticP (Supplementary Table S1). Further analysis of these candidates showed that 52% were predicted to be effector proteins, while 81% were predicted to be apoplastic proteins (Supplementary Table S1) (Sperschneider *et al*., 2016, 2018). The 90 candidate effector proteins were analysed using BLASTP (cut-off value as ≥50% identity and E-value ≤0.01) to search for homologues. Fifty-nine of these ZtSSP candidates had a homologue in another plant-pathogenic fungal species (Supplementary Tables S2, S7). Of the 59 ZtSSPs, 13 were found to have a homologue in *Zymoseptoria brevis* only (Supplementary Table S7). Forty-three ZtSSPs had a homologue in *Z. brevis* as well as another plant pathogen, while three had a homologue in a plant-pathogenic species other than Z. *brevis* (Supplementary Table S2). Of the 43 ZtSSPs, 17 were non-annotated proteins and 26 candidates had annotated protein domains. We examined the expression of these 17 conserved non-annotated effector candidates across three *Z. tritici* isolates at 7 dpi. The expression of Mycgr*3G105265* (*ZtSSP2*) was similar (FKPM 148, 126, and 177) across all three isolates (IPO323, 553.11, and 560.11) compared with other *ZtSSP* candidates (Supplementary Table S3). We selected the effector candidate ZtSSP2 for functional characterization as we reasoned that the conservation across different plant pathogenic species (Supplementary Tables S2, S5) and the similar levels of expression across three different *Z. tritici* isolates (Supplementary Table S3) may indicate a conserved core effector.

**Fig. 1.**
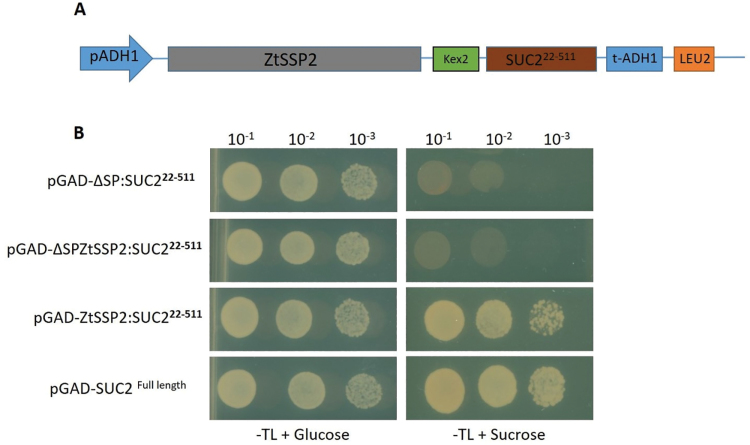
ZtSSP2 is a secreted protein. (A) Design of the Gateway-compatible yeast pGADT7-ZtSSP2-Suc2 vector and invertase mutant yeast strain SEY6210 used for the secretion assay (Brennan *et al.*, 2020). (B) The yeast strain carrying *ZtSSP2* with the secretion signal fused in-frame with the invertase gene Suc2 were able to grow in sucrose-containing drop out media (SD-TL), therefore cells will grow if invertase is secreted. SEY6210 carrying the pGAD-ΔSP:SUC2^22−5112^ vector was used as a negative control while SEY6210 with pGAD-SUC2^Full length^ acts as a positive control. This experiment was repeated three times independently with three replicates per independent experiment.

ZtSSP2 is predicted to have an N-terminal signal peptide (0.9986 likelihood, SignalP4.1) (Nielsen *et al.*, 2017). To validate this, we tested the secretion of ZtSSP2 using a yeast secretion assay (Fig. 1A; Brennan *et al.*, 2020). The full-length ZtSSP2 protein could complement the *suc2* knockout yeast strain allowing it to grow in selection media containing sucrose as the sole source of carbon (Fig. 1B). These characteristics suggest that ZtSSP2 is a conserved secreted effector protein.

### Homologues of ZtSSP2 are conserved in the Dothideomycete fungi

**Fig. 2.**
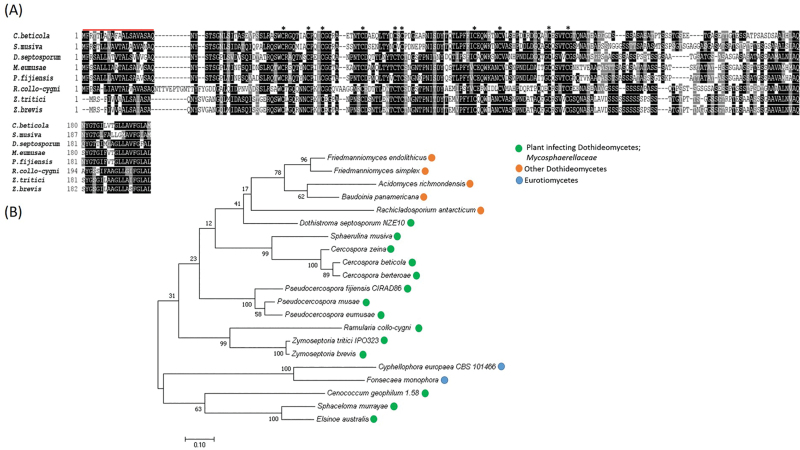
ZtSSP2 (*Z. tritici* small secreted protein candidate 2) homologues are widely present across Dothideomycetes. (A) Protein alignment using ClustalW of the full protein sequence of *Z. tritici* ZtSSP2 with homologues from seven plant-infecting Dothideomycetes fungi. Asterisks indicate conserved cysteine residues and shading represents identical or similar amino acids. Signal peptide predicted using SignalP4.1. (B) The unrooted maximum likelihood phylogeny of ZtSSP2 and the 20 closest orthologues including plant pathogens (green), other non-pathogenic Dothideomycetes (orange), and Eurotiomycetes (blue) which are human pathogens. The tree was generated with MEGA7 (Kumar *et al.*, 2016), Bootstrap values are based on 1000 replications. Sequences were obtained by blastp (NCBI) and aligned using ClustalW.

For ZtSSP2, we found additional potential homologues in other Dothideomycetes. All the potential homologues of ZtSSP2 identified within the plant pathogenic *Mycosphaerellaceae* family were of similar size, possessed an N-terminal signal peptide, and contained 10 conserved cysteine residues (Fig. 2A). For example, the homologue from the conifer-infecting fungi *Dothistroma septosporum* shares 53.57% sequence similarity, that from the barley pathogen *R. collo-cygni* is 51.67% identical to ZtSSP2, and that from the banana pathogen *Mycosphaerella fijiensis* shares 51% sequence identity. The majority of homologues (13) were from plant pathogens, but other homologues were from two Eurotiomycetes which are human pathogens (Fig. 2B; Supplementary Table S5). There was also an outgroup of Dothidoemycetes which were non-pathogenic (Fig. 2B).

### *ZtSSP* expression during STB infection in wheat

**Fig. 3.**
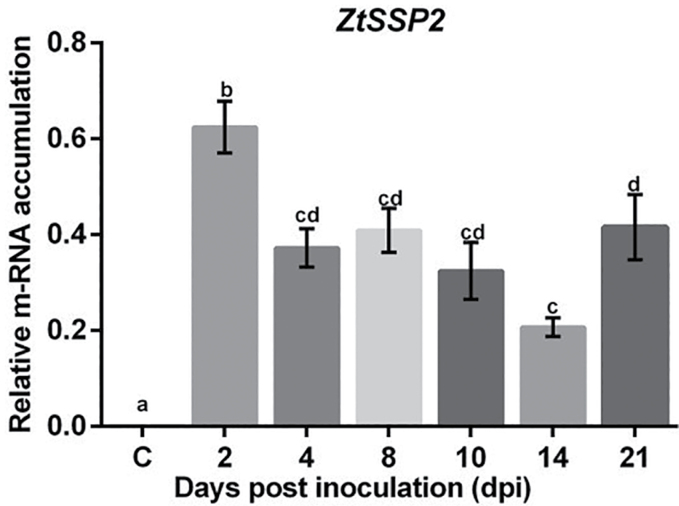
*ZtSSP2* is expressed at different stages of *Z. tritici* infection (2–10 dpi biotrophic stage and 14–21 dpi necrotrophic stage). Gene expression analysis of *ZtSSP2* uninfected control and at 2, 4, 8, 10, 14, and 21 dpi of wheat (cv. Remus) with *Z. tritici* isolate 560.11 (Lynch *et al.*, 2016). Control plants were inoculated with Tween-20. RNA was extracted from three wheat leaves per time point followed by reverse transcription into cDNA. The qPCR was performed on cDNA using specific primers for *ZtSSP2*. The expression levels of *β-tubulin* (*Z. tritici*) were used to normalize the expression levels of *ZtSSP2*. Each independent experiment had three leaves each from three individual plants. The bars represent the mean relative expression ±SEM of three independently replicated experiments. Different letters above bars indicate significant differences, as determined by Tukey’s test (*P<0.05).

Pathogen effector genes are known to be induced transcriptionally during infection of the host plant (Stergiopoulos and de Wit, 2009). We performed a quantitative reverse transcription–PCR (qRT–PCR) to determine the expression of *ZtSSP2* during infection. The *Z. tritici* isolate 560.11 (Lynch *et al.*, 2016) was used to infect wheat (cv. Remus) and the expression of *ZtSSP2* was determined over 2, 4, 8, 10 dpi (representing the biotrophic stage), 14, and 21 dpi (the necrotrophic stage) compared with the uninfected control (Fig. 3). Based on expression analysis, *ZtSSP2* was expressed from 2 dpi through to 21 dpi. The expression was significantly higher at 2 dpi compared with all other time points, suggesting a potential role early in biotrophy. There was a significant dip in expression at 14 dpi compared with 2 and 21 dpi (Fig. 3).

### *ZtSSP* candidates did not induce cell death in wheat

**Fig. 4.**
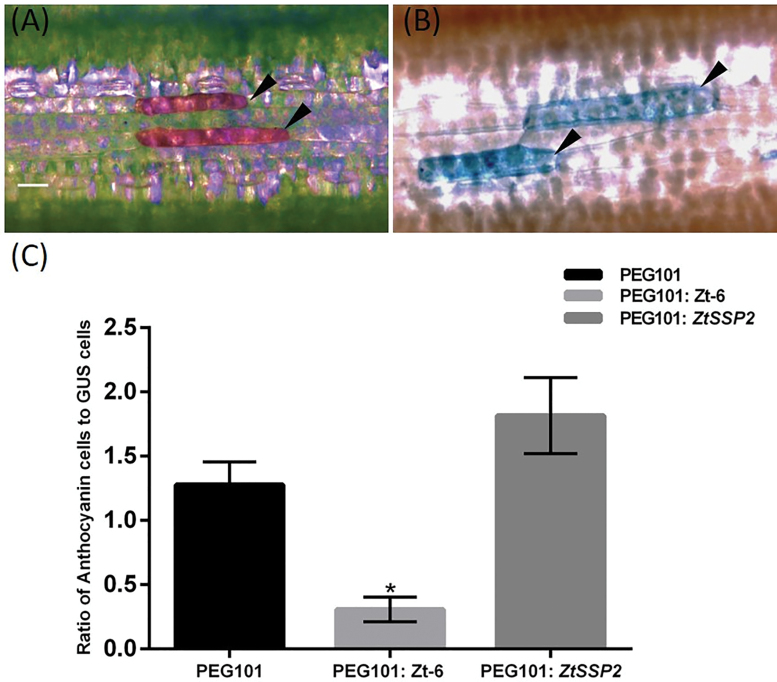
Single-cell death assay in wheat leaves. Wheat leaves of cv. Kanzler were co-bombarded with pUbiGus as a transformation marker, pEG101 for overexpression of *ZtSSP2* (PEG101:ZtSSP2), and the *B-Peru/C1*-expression plasmid pBC17 that induces anthocyanin accumulation in wheat epidermal cells. (A) Unstained wheat leaf showing epidermal cells that accumulate anthocyanin (arrow; scale bar=20 µm) 4 d after bombardment and (B) GUS-expressing cells after GUS staining. (C) Quantification of the relative number of anthocyanin-producing cells calculated as the ratio of anthocyanin-accumulated cells to the number of GUS-expressing cells. Co-transformation of pBC17 and pUbiGUS with the empty vector pEG101 and pEG101:*Zt-6* served as a positive control inducing cell death. Values are the means of four independent experiments with seven leaves counted per repetition (bars ±SEM). The asterisk on top of the bar represents significant differences determined by Tukey test (*P<0.05).

We used a transient leaf expression system developed by Pliego *et al.* (2013) to assess whether ZtSSP2 can induce cell death. In this system, transient expression of the maize transcription factor genes *B-Peru* and *C1* leads to accumulation of anthocyanin only in intact vacuoles of viable cells and can therefore be used as a cell death marker (Schweizer *et al.*, 2000). We co-bombarded the pEG101:(ΔSP) *ZtSSP2* overexpression construct with pUbiGUS (cell death-insensitive transformation marker) and the anthocyanin expression plasmid pBC17 into wheat leaves. Co-bombardment of pBC17 with pUbiGUS and the vector pEG101 was used as a negative control, while *Zt-6*, previously reported to induce cell death in wheat (Kettles *et al.*, 2018), was used as a positive control. The number of cells accumulating anthocyanin (Fig. 4A) was counted and then leaves were subsequently stained for GUS activity (Fig. 4B). The number of cells stained with GUS was also counted. The ratio of anthocyanin to GUS cells was used as an estimate of cell death. The empty vector control (pEG101) resulted in the average ratio of 1.27 (Fig. 4C), while the positive control (pEG101:*Zt-6*) showed a reduced ratio of 0.3 (*P*<0.05). The anthocyanin to GUS ratio obtained from bombardment of ZtSSP2 was not significantly different from the control, suggesting no cell death activation by this effector candidate in wheat.

### Candidate ZtSSP2 interacts with wheat ubiquitin ligase *in vitro* and *in planta*

**Fig. 5.**
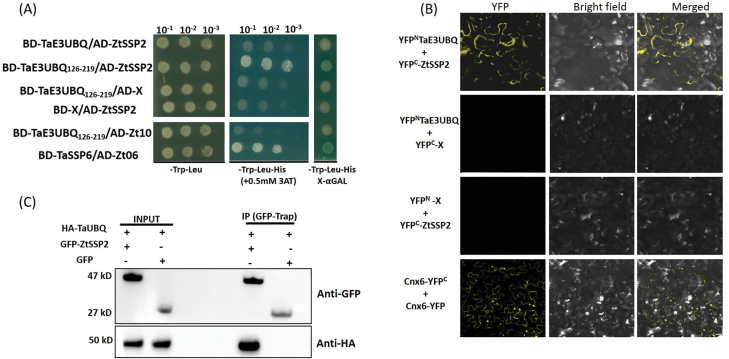
Interaction of (ΔSP) ZtSSP2 with host protein TaE3UBQ. (A) Yeast two-hybrid assay using the yeast cells transformed with *TaE3UBQ* and (ΔSP) *ZtSSP2* cloned in the Gal4 bait (BD) and prey vectors (AD). Yeast strain Y2HGold co-expressing the vector BD containing *TaE3UBQ*, *TaE3UBQ*_*126–219*_, *TaSSP6*, or empty bait vector (BD-X) and the prey vector containing *ZtSSP2*, *Zt10*, *Zt06*, or empty vector (AD-X) were grown on auxotrophic medium (SD/-Leu-Trp) (left panel) or selective Trp/Leu/His drop out medium in the presence of 0.5 mM 3-amino-1,2,4-triazole (3-AT). Only yeast cells co-expressing *ZtSSP2* and *TaE3UBQ*_*126–219*_ grew on selective medium (SD/-Leu-Trp-His) (middle panel) and showed α-galactosidase activity encoded by α-galactosidase (MEL1) (right panel). BD-TaSSP6 and AD-Zt06 were used as positive controls (Zhou *et al.*, 2020). The experiment was repeated independently three times, three plates per experiment with similar results. (B) Validation of *in planta* interaction of (ΔSP) *ZtSSP2* with wheat ubiquitin protein visualized by the BiFC assay. Confocal microscopy images of representative *N. benthamiana* epidermal leaf cells expressing proteins fused to the N- or C-terminal part of YFP as indicated. YFP and brightfield are shown both separately and as an overlay. Scale bar=10 μm. In both experiment (A) and (B), candidate Zt-10 was used as a negative control while Cnx6 homodimerization was used as a BiFC positive control. Experiments were repeated at least twice independently with similar results (three leaves each from individual plants). (C) Confirmation of the interaction between ZtSSP2 and TaE3UBQ by co-immunoprecipitation assays. Western blot of total proteins from *N. benthamiana* leaves co-infiltrated with the construct and co-immunoprecipitated using GFP-Trap magnetic beads. Expression of constructs in the leaves is indicated by ‘+’. Immunoblots were performed using anti-GFP and anti-HA antibodies. The protein size markers are indicated in kDa.

**Fig. 6.**
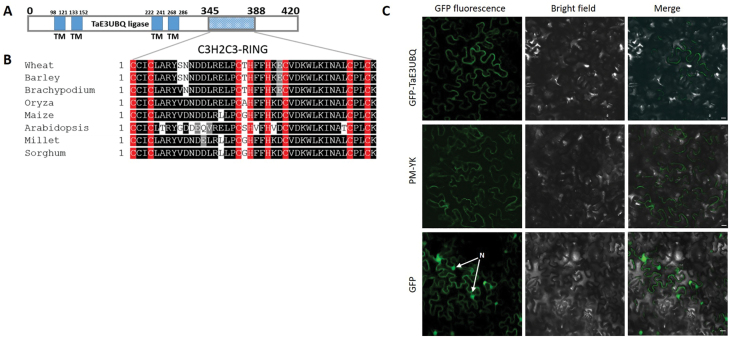
Sequence analysis of wheat E3 ubiquitin ligase [*T. aestivum* E3 UBQ ligase protein (TaE3UBQ)]. (A) Schematic representation of the structure of TaE3UBQ and the conserved RING finger motif using TMHMM v.2 and the CDD. (B) Sequence alignment of the C3H2C3-type RING finger conserved motif in TaE3UBQ homologues in barley (GenBank accession no. BAJ95361.1), *Brachypodium* (XP_003568812.1), *Oryza* (XP_015640584.1), maize (PWZ17186.1), Arabidopsis (NP_178156.1), millet (RLM97696.1), and sorghum (XP_002439378.1). Putative Zn^2+^-interacting amino acid residues are indicated in red, while conserved and non-conserved residues are highlighted in black and white, respectively. (C) GFP–TaE3UBQ fluorescent signal localizes to the cell periphery similar to the signal obtained from a plasma membrane marker pm-yk (Nelson *et al.*, 2007) in *N. benthamiana* cells. The GFP fluorescent protein was found distributed throughout the cell of transformed *N. benthamiana* leaf cells. Scale bars=20 µm

We performed a yeast two-hybrid screen to determine if ZtSSP2 could interact with wheat host components. A cDNA expression library generated from *Z. tritici*-infected wheat leaves was screened using ZtSSP2 as bait. A wheat cDNA clone encoding the extracellular region (amino acids 126–219) of a C3H2C3-type RING E3 ubiquitin ligase protein (TaE3UBQ) was identified. Pathogen effectors have been reported to target the host ubiquitin system to manipulate host defence (Park *et al.* 2012). To investigate if ZtSSP2 interacts with the wheat E3 ligase (TaE3UBQ) and thereby manipulates the wheat host ubiquitin–proteasome system (UPS), this protein–protein interaction was tested in yeast (*Saccharomyces cerevisiae*) using a galactose-responsive transcription factor GAL4-based yeast two-hybrid system (Fig. 5A). We cloned TaE3UBQ and TaE3UBQ_126–219_ from wheat cDNA and found that (ΔSP)ZtSSP2 interacted with TaE3UBQ_126–219_ but not with the full-length protein in yeast (Fig. 5A). The TaE3UBQ homologue HvE3UBQ was found in barley, and the ZtSSP2 homologue RcSSP2 was found in the barley pathogen *R. collo-cygni* (Fig. 6). Therefore, this interaction was also tested (Supplementary Fig. S4). However, no positive interaction between (ΔSP)RcSSP2 and HvE3UBQ was observed. When RcSSP2 was replaced with (ΔSP)ZtSSP2, we found that HvE3UBQ_126–219_ also interacts with ZtSSP2 in yeast (Fig. S4).

Additionally, the interaction between (ΔSP)ZtSSP2 and TaE3UBQ was investigated *in planta* using the BiFC system (Gehl *et al.*, 2009). TaE3UBQ and (ΔSP)ZtSSP2 were fused at the N- and C-terminus, respectively, of YFP. The resulting constructs were co-expressed using *Agrobacterium* infiltration in *N. benthamiana* leaves. A strong YFP signal was observed when YFP^N^–TaE3UBQ was co-infiltrated with YFP^C^– (ΔSP)ZtSSP2 (Fig. 5B). To test if the interaction of TaE3UBQ was specific to ZtSSP2, co-infiltration with another putative *Z. tritici* effector, YFP^C^–(ΔSP)Zt-10, was performed. No YFP fluorescence was observed with this construct, suggesting no interaction of TaE3UBQ with candidate (ΔSP)Zt-10. Cnx6 homodimers were used as a positive control (Gehl *et al.*, 2009).

We used Co-IP to further validate the interaction of TaE3UBQ with ZtSSP2. The HA-tagged TaE3UBQ was co-expressed with a GFP fusion of ZtSSP2 in *N. benthamiana* and subjected to Co-IP assay using GFP-Trap^®^-M magnetic beads. Western blot analysis showed that only HA-tagged TaE3UBQ was co-immunoprecipitated on GFP-Trap^®^-M beads and specifically detected with the anti-HA antibody in the presence of GFP–ZtSSP2, but not in the GFP control (Fig. 5C). Taken together, these results reveal that ZtSSP2 interacts with TaE3UBQ *in vitro* and *in planta*.

### Wheat ubiquitin represents is a C3H2C3 ring finger E3 protein ligase with a transmembrane helix

The full-length cDNA of TaE3UBQ (TraesCS1D02G119700) was obtained by comparing the clone sequence with the EnsemblPlants IWGSC database. BLASTp showed that TaE3UBQ has two additional homeologues in wheat, namely TraesCS1A02G118800 and TraesCS1B02G138300, sharing 99.3% and 97% similarity with its 1D variant, respectively. The TaE3UBQ ORF encodes a RING finger protein of 420 amino acids, with a theoretical pI value of 6.06 and a deduced molecular mass of 46.2 kDa (Fig. 6A). Protein sequence analysis using NCBI CDD (Marchler-Bauer *et al.*, 2011) and MemBrain 3.1 (Yin *et al.*, 2018) programs showed a C-terminal C3H2C3 zinc-finger domain with a transmembrane helix and extracellular loop region (Fig. 6A, B; Supplementary Fig. S5). Alignment of TaE3UBQ revealed that the RING finger domain is conserved among various plant species (Fig. 6B). We performed localization of GFP-tagged TaE3UBQ in *N. benthamiana* leaves. The fluorescent signal from *GFP-TaE3UBQ* was predominantly localized at the cell periphery similar to the signal obtained by expression of a plasma membrane marker pm-yk (Nelson *et al.*, 2007), whereas the GFP control was distributed throughout the cell including the nucleus (Fig. 6C).

### Silencing *TaE3UBQ* enhances wheat susceptibility to *Z. tritici*

**Fig. 7.**
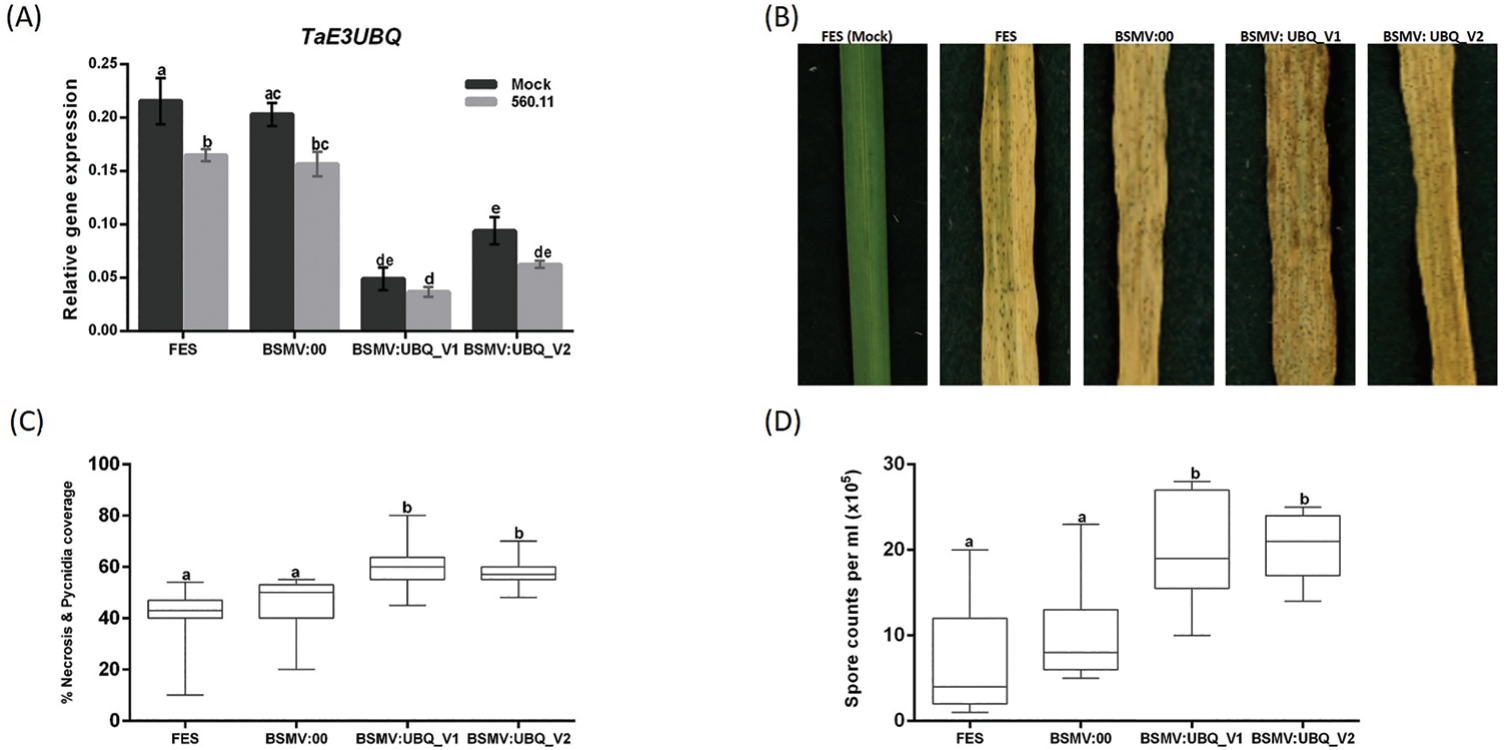
BSMV-mediated virus-induced gene silencing (VIGS) of the wheat E3 ubiquitin ligase gene (*TaE3UBQ*) resulted in increased Z*. tritici* susceptibility of wheat leaves. Wheat leaves at the second leaf stage (cv. Longbow) were treated with either FES (VIGS buffer), BSMV:00 (empty vector), or BSMV:UBQ_V1 or BSMV:UBQ_V2 (constructs targeting all three homeologues of *TaE3UBQ*). The third and fourth leaves of VIGS-treated plants were inoculated with either Tween (mock) or *Z. tritici* (560.11). Subsequently, the third leaves were collected for gene expression studies and the fourth leaves for phenotyping and spore count. (A) Relative transcript abundance of all *TaE3UBQ* homeologues in knockdown plants (5–6 leaves each from individual seedlings per treatment per replicate). (B and C) Phenotype of VIGS-treated leaves infected with *Z. tritici* at 21 dpi (10 leaves each from individual seedlings per treatment per replicate). (D) Average spore counts per millilitre were counted over 2 cm leaf lengths (seven leaves each from individual seedlings per treatment per replicate). Three independent experiments were carried out. Bars with the same letter are not significantly different. (*P*<0.05).

We examined the expression of *TaE3UBQ* homeologues using expVIP (expression Visualization and Integration Platform) (Borrill *et al.*, 2016). All three wheat homeologues have a similar expression profile and levels were not significantly different between mock and *Z. tritici* treatment (Supplementary Fig. S6). VIGS was used to determine the role of *TaE3UBQ* during the interaction of *Z. tritici* with wheat (cv. Longbow) (Fig. 7). Two independent non-overlapping constructs (UBQ_V1and UBQ_V2) were used to target all three *TaE3UBQ* homeologues (Supplementary Fig. S3).

Expression of three homeologues was measured using a primer pair in a conserved region (Supplementary Fig. S3). qRT–PCR analysis of gene-silenced leaf tissue (BSMV:UBQ_1 and BSMV:UBQ_2) showed that, compared with the control (BSMV:00) plants, *TaE3UBQ* expression (all homeologues) was significantly reduced by 53–70% in mock-inoculated leaves and by 59–76% in *Z. tritici*-inoculated leaves of the wheat cv. Longbow (Fig. 7A). Silencing using construct UBQ_V1 showed higher efficiency than construct UBQ_V2. Phenotypic assessment of wheat leaves at 21 d post-*Z. tritici* infection revealed that BSMV:UBQ_V1 and BSMV:UBQ_V2 leaves had significantly increased disease coverage as represented by higher necrosis and pycnidia coverage compared with control leaves (BSMV:00) (Fig. 7B, C). This was reflected by the significantly higher *Z. tritici* spore numbers found in BSMV:UBQ_V1- and BSMV:UBQ_V2-treated leaves compared with the BSMV:00 control (Fig. 7D).

## Discussion

The major components of the filamentous plant pathogens secretome are often small secreted cysteine-rich proteins (SSPs) (Stergiopoulos and de Wit, 2009). These SSPs are small (300 amino acids) with cysteine residues and a secretion signal at the N-terminus. SSPs from plant pathogens play a key role in subverting host plant immunity and facilitating colonization (Hogenhout *et al.*, 2009; Rafiqi *et al.*, 2013). Therefore, understanding how pathogen SSPs function in a host plant and the potential host targets is key for complete understanding of the molecular mechanism of pathogenicity and disease.

In this study, we characterize a small secreted protein ZtSSP2, as an effector candidate, from *Z. tritici* which is conserved across the Dothideomycetes. Fungal pathogen effectors are secreted in the host, have diverse functions, and are differentially regulated throughout infection (Chen *et al.*, 2013). ZtSSP2 was functionally secreted using a yeast secretion system, suggesting it is a potential pathogen secreted effector candidate protein (Fig. 1).

*Zynoseptoria tritici* has a long latent/biotrophic phase prior to necrotrophy and has been described as a hemibiotroph (Rudd *et al.*, 2015) or a latent necrotroph (Sánchez-Vallet *et al.*, 2015). During the latent phase which lasts for 7–10 d, *Z. tritici* may be endophytic (Sánchez-Vallet *et al.*, 2015) or even epiphytic (Fones *et al.*, 2017). *ZtSSP2* expression levels were highest at 2 dpi during the latent/biotrophic phase of the pathogen before dipping at 14 dpi following the switch to the necrotrophic phase which takes place from around 10 dpi (Rudd *et al.*, 2015). A biphasic expression pattern was also reported for *ZtSSP2* previously (Mirzadi Gohari *et al.*, 2015).

To test a possible role for ZtSSP2 in cell death and potentially the necrotrophic lifestyle of *Z. tritici*, we utilized a biolistic approach with an anthocyanin marker for cell death in wheat. Our results showed that ZtSSP2 does not induce cell death in wheat leaves. Ubiquitin E3 ligases can act as either positive or negative regulators of plant immunity controlling the degradation of different protein substrates (McLellan *et al.*, 2020). In rice, the RING E3 ligases APIP6 and APIP10 are positive regulators of PTI as well as targets of the *Magnaporthe oryzae* effector AvirPiz-t. When either APIP6 or APIP10 is silenced, PTI is compromised, including impaired reactive oxygen species production and defence gene induction (Park *et al.*, 2016). The reactive oxygen species hydrogen peroxide is known to restrict *Z. tritici* growth during the latent/biotrophic phase (Shetty *et al.*, 2003). It is conceivable that TaE3UBQ plays a similar role in wheat, positively regulating PTI. Overexpression of *ZtSSP2 in planta* may promote interaction with TaE3UBQ and compromise PTI which would not induce cell death, in agreement with our results (Fig. 4). Silencing TaE3UBQ would also compromise PTI. This is in line with the increased necrosis and pycnidia formation observed with VIGS of TaE3UBQ (Fig. 7). Thus, ZtSSP2 may interact with TaE3UBQ to compromise E3UBQ ligase activity, suppressing PTI.

ZtSSP2 has homologues in other members of the Dothideomycetes class, including the pine-infecting hemibiotroph *D. septosporum*, banana-infecting *P. fijiensis*, and the barley pathogen *R. collo-cygni*. This broad conservation suggests that ZtSSP2 may be a core effector candidate in Dothideomycete pathogens. Of the homologous proteins identified, 13 of these are from plant pathogens (Fig. 2B). We explored a possible interaction between *R. collo-cygni* RcSSP2 and barley HvE3UBQ. However, we did not observe a strong interaction of RcSSP2 (homologue of ZtSSP2 with 51.67% similarity) with HvE3UBQ or HvE3UBQ_126–219_; however, ZtSSP2 was found to interact with HvE3UBQ_126–219_ (Supplementary Fig. S4), demonstrating that this plant host target is conserved. Homologues do also exist in two Eurotiomycetes (*Cyphellophora europaea* and *Fonsecaea monophora*; 40–45% homology) which can cause disease in humans (de Hoog *et al.*, 2000; Queiróz *et al.*, 2018). In the outgroup of other Dothideomycetes which are non-pathogenic (Fig. 2B), some of these have been reported to be lichen-forming fungi. Recently, lichen-forming fungi were reported to express SSPs (Armaleo *et al.*, 2019).

A range of virulence effectors from plant pathogens concentrate into a limited number of host cellular target ‘hubs’ to subvert the host defence and enhance virulence (Mukhtar *et al.*, 2011). One of the key regulatory networks in plant defence is the UPS. This UPS regulates multiple aspects of plant immunity involving recognition, receptor protein accumulation, and subsequent defence signalling (Marino *et al.*, 2012; Ustun *et al*., 2016). Therefore, manipulation of the host UPS by effectors is central to increasing pathogen virulence. Here, the *Z. tritici* candidate effector ZtSSP2 was found to physically interact with a wheat host E3 ubiquitin ligase (TaE3UBQ) both in yeast and *in planta*. Domain analysis of TaE3UBQ showed that it possesses a conserved RING-finger domain and four transmembrane domains accompanied by an extracellular loop in the middle (Fig. 6A; Supplementary Fig S5). The localization of *TaE3UBQ* in *N. benthamiana* leaves suggests that TaE3UBQ could be localized predominantly to the cell periphery as a membrane-localized protein. RING-finger domains are characteristic of RING-class E3 ubiquitin protein ligases that transfer ubiquitin from an E2 enzyme to a substrate protein. The RING domain mediates the interaction with the appropriate E2 enzyme (Van Wijk *et al.*, 2009). These E3 ligases are central to plant immune responses and are also known targets for pathogen-secreted effector proteins. One such example was effector *AvrPiz-t* from the blast fungus *M. oryzae. AvrPiz-t* has been shown to interact with and inhibit the rice RING-type E3 ubiquitin ligase (APIP6) *in vitro*, resulting in the suppression of the APIP6-mediated PTI response (Park *et al.*, 2012). The effector AVR3a from *P. infestans* interacts with and stabilizes the host U-box E3 ligase CMPG1 (Cys, Met, Pro, and Gly protein 1) that is required for INF-1-triggered cell death. The Avr3a interaction with CMPG1 leads to CMPG1 modification and thus prevents host cell death induction during infection (Bos *et al.*, 2010).

In wheat, we found three homoalleles of the *E3UBQ* gene on chromosome 1DS, 1A, and 1B, and we hypothesize that all of them could be targets of ZtSSP2 as they appear to be conserved (Supplementary Fig. S6). For example, this was also observed for the stripe rust effector PEC6 that interacts with wheat adenosine kinases to suppress wheat defence (Liu *et al.*, 2016). To understand the role of *TaE3UBQ* during *Z. tritici* infection, we silenced the gene transcript using BSMV VIGS. Silencing of *TaE3UBQ* resulted in increased *Z. tritici* symptoms. We speculate that ZtSSP2 binding to *TaE3UBQ* suppresses the wheat ubiquitin system and PTI. There exists accumulating evidence that E3 ubiquitin ligase is a central regulator of plant immunity and signalling (Trujillo and Shirasu, 2010; Marino *et al.*, 2012). In rice, the resistance gene *Xa21* (*Xanthomonas oryzae* pv. *oryzae* locus 21) was shown to require a RING-E3 ubiquitin XA21-binding protein 3 (XB3) which plays a key role in accumulation of the XA21 protein and Xa21-mediated disease resistance (Wang *et al.*, 2006). In Arabidopsis, the Plant U-Box 12 (PUB12), a U-box E3 ligase, is involved in the PTI response against bacterial flagellin through Flagellin sensing 2 (FLS2) (Lu *et al.*, 2011). Similarly, the Arabidopsis Tóxicos en Levadura (ATL) family of RING finger E3 ligase (ATL9) is induced by fungal chitin and is involved in resistance against the biotrophic fungal pathogen, *Golovinomyces cichoracearum* (Deng *et al.*, 2017).

In conclusion, the ZtSSP–TaE3UBQ interaction may modulate and compromise E3UBQ ligase activity suppressing wheat PTI. VIGS of TaE3UBQ was found to promote STB susceptibility, which agrees with TaE3UBQ being a positive regulator of PTI. However, further work is needed to explore the outcome of ZtSSP2 interaction on TaE3UBQ activity and to identify downstream host interactors of TaE3UBQ which will provide information on how TaE3UBQ might regulate immunity in wheat.

## Supplementary data

The following supplementary data are available at *JXB* online.

Fig. S1. *In silico* selection of non-annotated small secreted proteins (ZtSSPs) of *Z. tritici*. 

Fig. S2. BSMV-mediated gene silencing (VIGS) of the *phytoene desaturase* (*PDS*) gene in wheat.

Fig. S3. Three homologues of *TaE3UBQ* are of high similarity. 

Fig. S4. Yeast two-hybrid assay to test the interaction of RcSSP2 with HvE3UBQ.

Fig. S5. TaE3UBQ protein topology illustration using MemBrain 3.1 (Yin *et al.*, 2018). 

Fig. S6. Expression profiles of wheat E3 ubiquitin ligase homeologues during *Z. tritici* infection. 

Table S1. List of putative candidate effector proteins of *Z. tritici* (*ZtSSP*s). 

Table S2. *Z. tritici* small, secreted proteins have potential homologues in other plant pathogens.

Table S3. Expression of 17 conserved effector candidates across three *Z. tritici* isolates IPO323, 553.11, and 560.11 at 7 dpi. 

Table S4. List of primers used in this study.

Table S5. ZtSSP2 homologues in the Dothideomycetes with species, accession numbers, description, e-value, sequence, and length of the 20 closest homologues using NCBI BlastP.

Table S6. List of wheat proteins identified as a potential interactors with *Z. tritici* candidate ZtSSP2. 

Table S7. *Z. tritici* small secreted proteins with potential homologues only present in *Zymoseptoria brevis*.

eraa489_suppl_Supplementary_Figure

eraa489_suppl_Supplementary_Table

## Data Availability

All data supporting the findings of this study are available within the paper. *ZtSSP* expression (corresponding to Supplementary Table S1) are available at the Dryad Digital Repository (https://doi.org/10.5061/dryad.9w0vt4bcx; Karki *et al.*, 2020).
