# Supplementary material for: A small secreted protein from Zymoseptoria tritici interacts with a wheat E3 ubiquitin ligase to promote disease
Source: J Exp Bot. 2020 Oct 23;72(2):733–46. doi: 10.1093/jxb/eraa489 (PMC7853600; doi:10.1093/jxb/eraa489)
Supplement: eraa489_suppl_Supplementary_Figure [file eraa489_suppl_supplementary_figure.pdf]

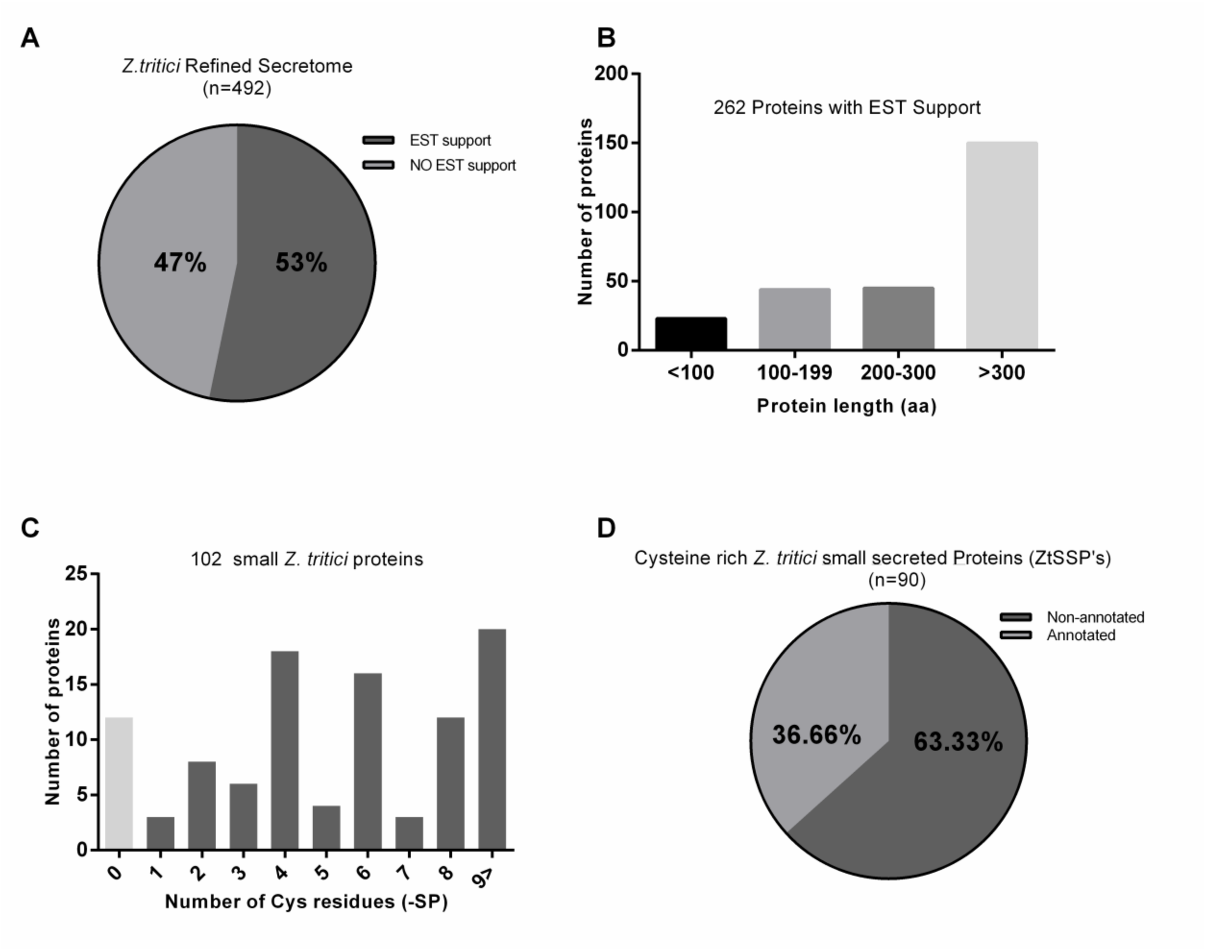

Fig. S1 *In silico* selection of non-annotated small secreted proteins (ZtSSP) of *Z.tritici*. (a) Selection of small proteins, 492 proteins encoded by the *Z. tritici* genome with EST support (do Amaral et al., 2012). (b) Proteins were classified based on their size and proteins with 50-315 amino acid length were selected. (c) Numbers of cysteine residues in the selected mature proteins after removing signal peptide. (d)Proteins were annotated based on the presence of conserved functional domain.

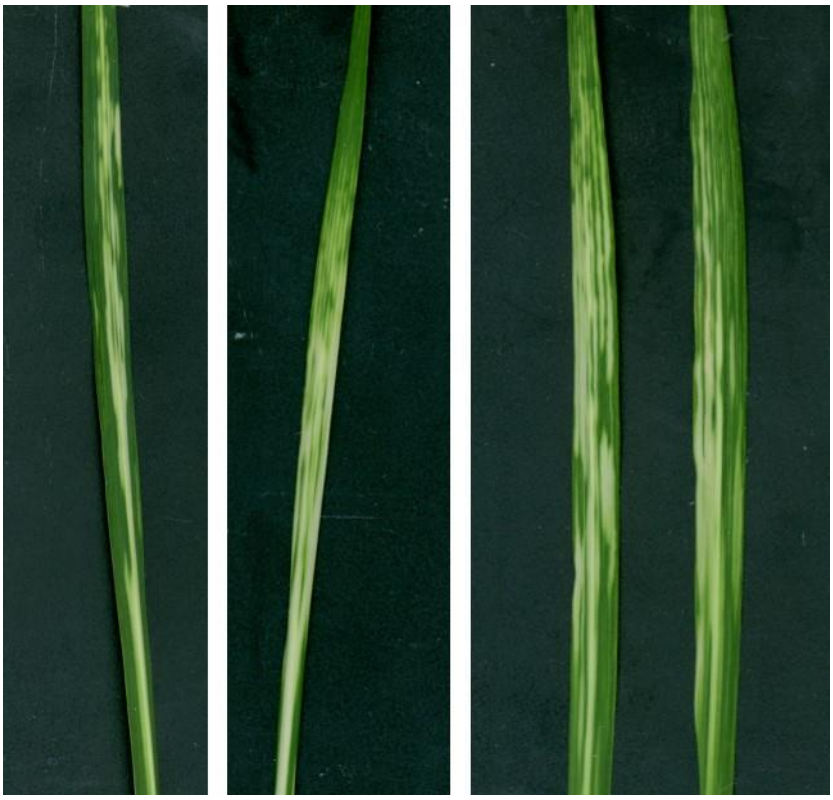

Fig. S2 Barley Stripe Mosaic Virus (BSMV) mediated gene silencing (VIGS) of *phytoene desaturase* (*PDS*) gene in wheat. Image of bleached PDS silenced fourth leaf at 10-14 days post inoculation with BSMV.

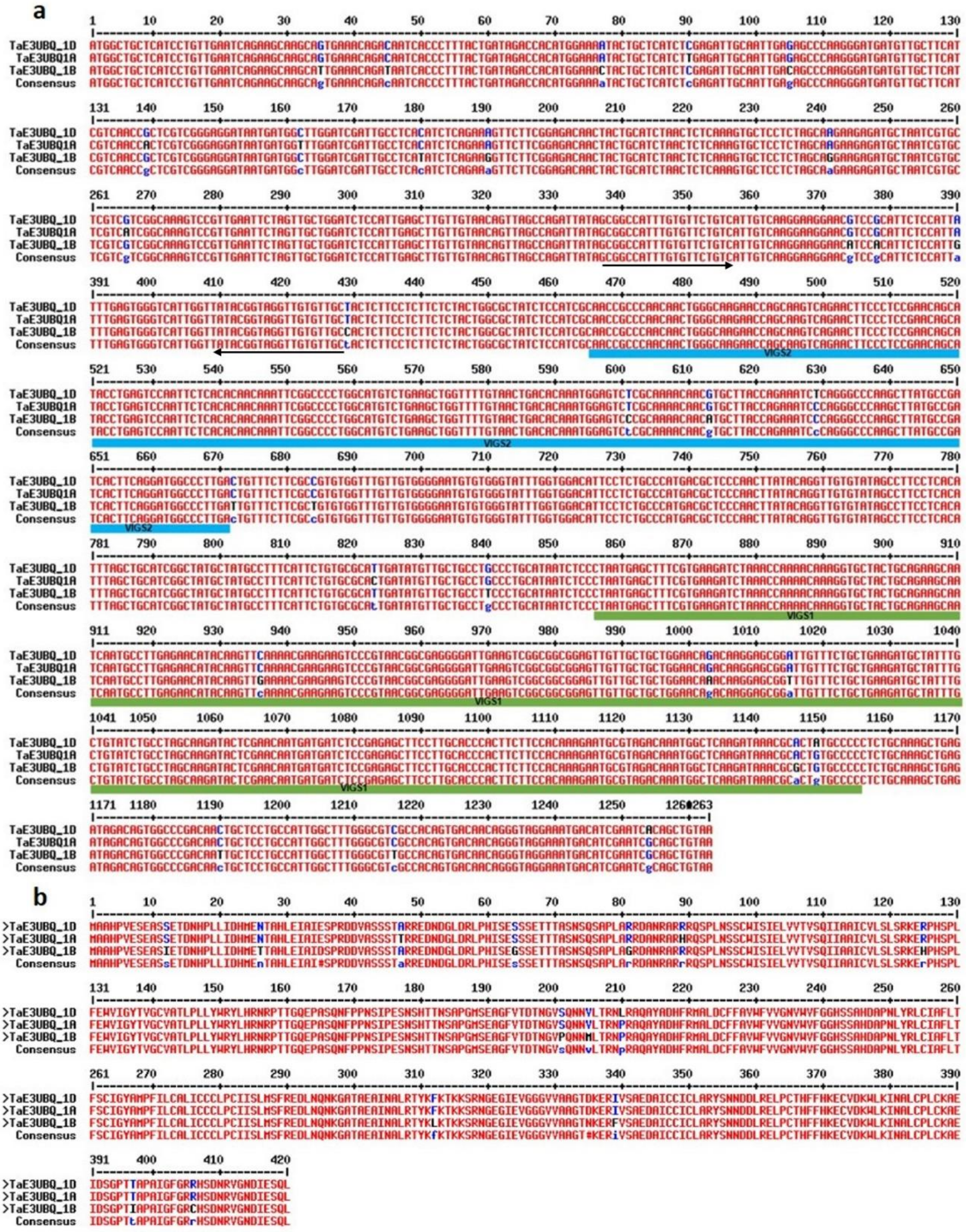

Fig. S3 Three homologues of *TaE3UBQ* are of high similarity. a) Nucleotide sequence alignments and (b) Amino acid sequences of three homologues of *TaE3UBQ* gene were obtained from Ensembl plant database. The three homologues are located on chromosomes 1A, 1B and 1D, respectively, base variations indicated with blue. The sequence alignment was performed using *MultAlin* (Corpet, 1998). Regions targeted by VIGS1 and VIGS2 constructs are highlighted below by a solid blue and green line, respectively. Region targeted by conserved qPCR primers are shown by black arrows.

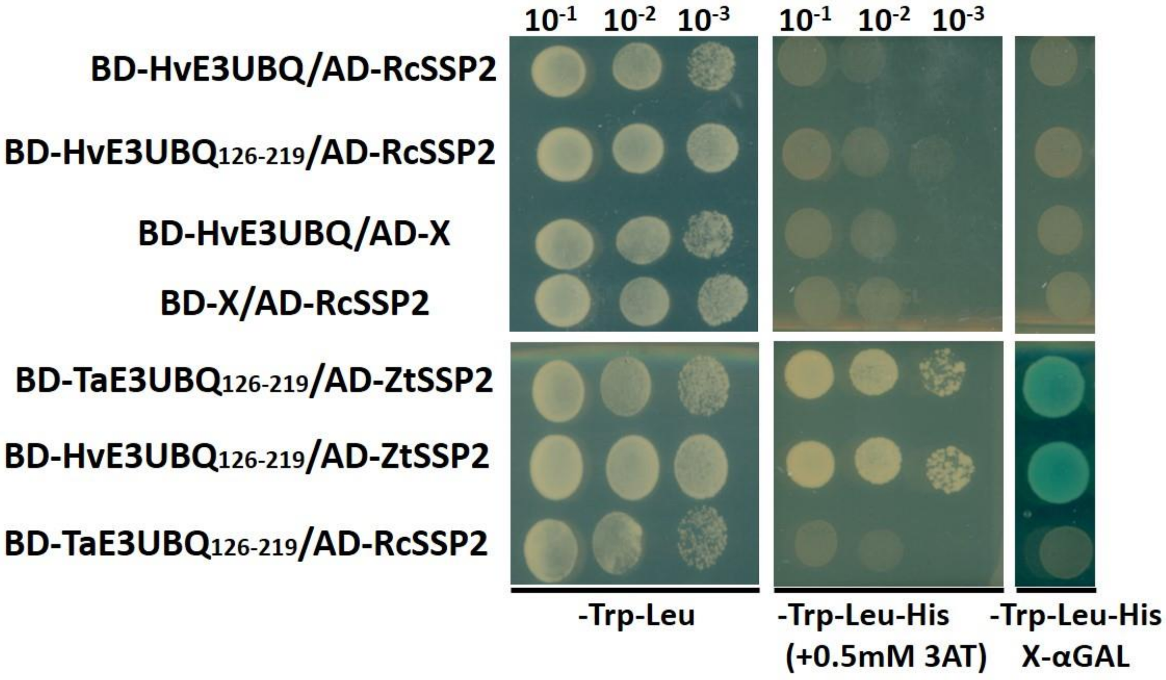

Fig. S4 Yeast two hybrid assay to test the interaction of *Ramularia Collo-cygni* homologue of ZtSSP2; RcSSP2 with Barley homologue of TaE3UBQ; HvE3UBQ. Yeast transformed with HvE3UBQ, HvE3UBQ126-219 and RcSSP2 cloned in the Gal4 bait and prey vectors. Yeast were grown for 4-5 days under selective Trp/Leu/His drop out medium in presence of 0.3mM 3-amino-1,2,4-triazole (3-AT) and X-α-gal medium or a non-selective Trp/Leu drop out medium (-TL) conditions. BD represents Binding domain & AD, activating domain. Experiment was repeated three times independently with three replicates per independent experiment.

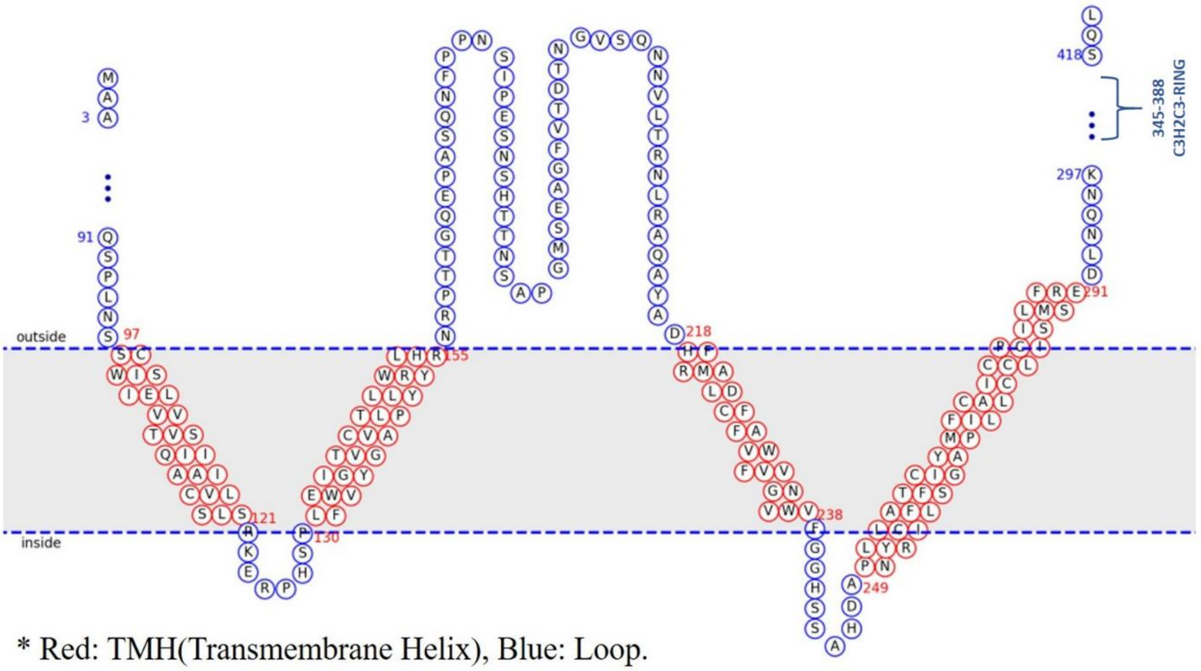

Fig. S5 TaE3UBQ protein topology illustration using MemBrain 3.1 (Yin *et al*., 2017). The protein sequence of TaE3UBQ shows transmembrane helix domains, an extracellular loop (155-218) and intracellular regions within the protein structure. The C3H2C3-ring is shown.

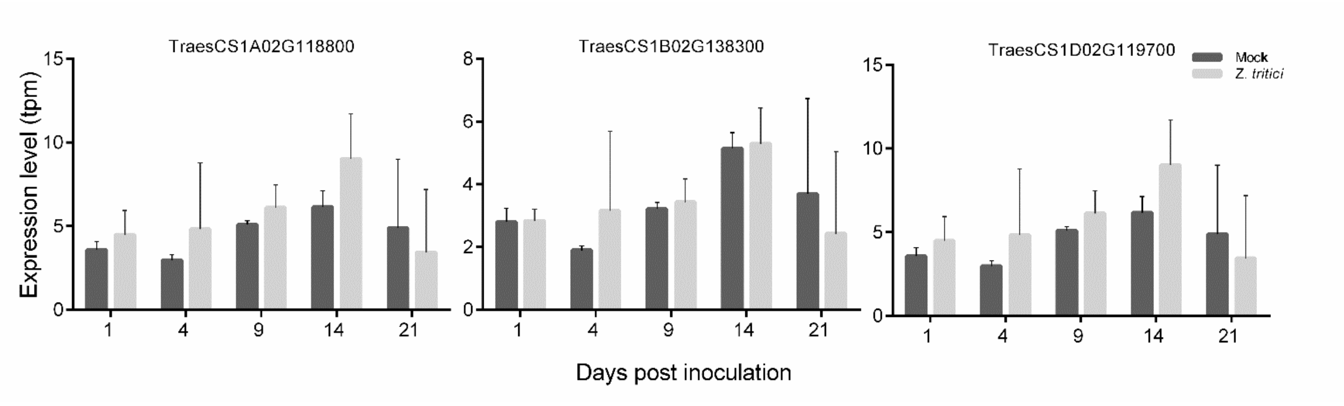

Fig. S6 Expression profiles of wheat E3 ubiquitin ligase homeologs during *Z. tritici* infection. Normalised expression data (in transcript per million (TPM)) retrieved from expVIP (Borrill *et al*., 2016) for all three TaE3UBQ homeologs following *Z. tritici* infection and mock treatment at 1, 4, 9, 14 and 21 days post inoculation (dpi).

Table S1. List of putative candidate effector proteins of *Z. tritici* (*ZtSSPs*).
Gene ID corresponds to JGI gene ID, Gene annotations were obtained from JGI database and blast search performed with NCBI BlastP. Functional annotation was performed using NCBI CDD database. EffectorP 1.0 and ApoplasticP 1.0 was used to predict if small, secreted proteins are predicted effectors and their localisation respectively: '+'and '-' indicates positive and negative prediction. Candidates that matched Kettles *et al.*, 2017 are referred as Zt.

**Codes**	**Gene ID JGI**	**Size (aminoacids)**	**Gene functional annotation (CDD database)**	**EffectorP 2.0 Prediction**	**ApoplasticP Prediction**
ZtSSP1	*Mycgr3G73448*	180	Hypothetical	+	-
ZtSSP2	*Mycgr3G105265*	200	Hypothetical	-	+
ZtSSP3	*Mycgr3G81079*	258	Hypothetical	+	-
ZtSSP4	*Mycgr3G103091*	83	Hypothetical	-	+
ZtSSP5	*Mycgr3G99161*	165	Hypothetical	+	+
ZtSSP6	*Mycgr3G100649*	76	Hypothetical	+	+
ZtSSP7	*Mycgr3G102617*	158	Hypothetical	+	+
ZtSSP8	*Mycgr3G103900*	130	Hypothetical	+	+
ZtSSP9	*Mycgr3G104000*	181	Hypothetical	+	-
ZtSSP10	*Mycgr3G104404*	180	Hypothetical	-	+
ZtSSP11	*Mycgr3G104444*	80	Hypothetical	+	+
ZtSSP12	*Mycgr3G104794*	158	Hypothetical	+	-
ZtSSP13	*Mycgr3G105182*	144	Hypothetical	+	+
ZtSSP14	*Mycgr3G105223*	189	Hypothetical	+	+
ZtSSP15	*Mycgr3G105659*	183	Hypothetical	-	-
ZtSSP16	*Mycgr3G105826*	99	Hypothetical	+	+
ZtSSP17	*Mycgr3G105896*	193	Hypothetical	+	-
ZtSSP18	*Mycgr3G106445*	120	Hypothetical	+	+
ZtSSP19	*Mycgr3G108482*	109	Hypothetical	+	+
ZtSSP20	*Mycgr3G110220*	132	Hypothetical	+	+
ZtSSP21	*Mycgr3G111008*	220	Hypothetical	-	+
ZtSSP22	*Mycgr3G111382*	191	Hypothetical	-	+
ZtSSP23	*Mycgr3G68477*	315	Hypothetical	-	+
ZtSSP24	*Mycgr3G90001*	287	Hypothetical	-	+
ZtSSP25	*Mycgr3G101652*	77	Hypothetical	+	+
Zt-4	*Mycgr3G104697*	150	Hypothetical	-	+
Zt-10	*Mycgr3G111505*	198	Hypothetical	-	+
Zt-14	*Mycgr3G107286*	117	Hypothetical	+	+
ZtSSP26	*Mycgr3G102996*	164	Hypothetical	+	+
ZtSSP27	*Mycgr3G103254*	101	Hypothetical	+	+
ZtSSP28	*Mycgr3G104758*	119	Hypothetical	+	+
ZtSSP29	*Mycgr3G104867*	171	Hypothetical	-	+
ZtSSP30	*Mycgr3G105677*	199	Hypothetical	+	+
ZtSSP31	*Mycgr3G106106*	157	Hypothetical	-	+
ZtSSP32	*Mycgr3G106345*	164	Hypothetical	-	+
ZtSSP33	*Mycgr3G106502*	90	Hypothetical	+	+
ZtSSP34	*Mycgr3G107824*	137	Hypothetical	-	+
ZtSSP35	*Mycgr3G108329*	193	Hypothetical	+	-
ZtSSP36	*Mycgr3G108877*	112	Hypothetical	+	+
ZtSSP37	*Mycgr3G110052*	180	Hypothetical	+	-
ZtSSP38	*Mycgr3G110144*	222	Hypothetical	-	+
ZtSSP39	*Mycgr3G41315*	196	Hypothetical	-	-
ZtSSP40	*Mycgr3G70376*	203	Hypothetical	-	+
ZtSSP41	*Mycgr3G95672*	142	Hypothetical	+	+
ZtSSP42	*Mycgr3G99124*	113	Hypothetical	+	+

ZtSSP43	*Mycgr3G79783*	184	Hypothetical	-	+
ZtSSP44	*Mycgr3G99676*	168	Hypothetical	-	+
ZtSSP45	*Mycgr3G83081*	53	Hypothetical	+	+
ZtSSP46	*Mycgr3G82925*	57	Hypothetical	+	+
ZtSSP47	*Mycgr3G81208*	59	Hypothetical	+	+
ZtSSP48	*Mycgr3G79286*	63	Hypothetical	+	+
ZtSSP49	*Mycgr3G79161*	68	Hypothetical	+	+
ZtSSP50	*Mycgr3G78893*	204	Hypothetical	-	+
ZtSSP51	*Mycgr3G106125*	70	Hypothetical	+	+
ZtSSP52	*Mycgr3G104383*	74	Hypothetical	+	+
ZtSSP53	*Mycgr3G83064*	75	Hypothetical	+	+
ZtSSP54	*Mycgr3G108329*	192	Hypothetical	+	-
ZtSSP55	*Mycgr3G67799*	273	Zinc peptidase	-	+
ZtSSP56	*Mycgr3G103792*	197	PAN domain	+	+
ZtSSP57	*Mycgr3G63409*	253	PEBP domain	-	+
ZtSSP58	*Mycgr3G67060*	267	Peroxidase_2	-	-
ZtSSP59	*Mycgr3G104571*	230	PEBP domain	-	-
ZtSSP60	*Mycgr3G105030*	269	Peptidase_A4	-	+
ZtSSP61	*Mycgr3G81448*	97	COesterase	-	+
ZtSSP62	*Mycgr3G105871*	246	Glyco_hydro_12	+	+
ZtSSP63	*Mycgr3G107289*	247	DUF3238	-	-
ZtSSP64	*Mycgr3G105487*	97	LysM	+	+
ZtSSP65	*Mycgr3G111027*	151	Alt_A1 superfamily	+	-
ZtSSP66	*Mycgr3G111636*	159	Hce2	+	+
ZtSSP67	*Mycgr3G109710*	200	Cap superfamily	+	+
ZtSSP68	*Mycgr3G99331*	224	Cutinase	-	+
ZtSSP69	*Mycgr3G100955*	254	UreF superfamily	-	-
ZtSSP70	*Mycgr3G102956*	258	Sod_Cu	-	+
ZtSSP71	*Mycgr3G74453*	296	LamG superfamily	-	+
ZtSSP72	*Mycgr3G93903*	297	Glyco_hydro_cc	-	+
ZtSSP73	*Mycgr3G106335*	238	CHRD domain	-	+
ZtSSP74	*Mycgr3G107904*	170	Hce2	+	+
ZtSSP75	*Mycgr3G108976*	200	NADHdh	-	+
ZtSSP76	*Mycgr3G68483*	216	Cutinase	-	+
ZtSSP77	*Mycgr3G77282*	231	Cutinase	+	+
ZtSSP78	*Mycgr3G71724*	273	Glyco_hydro_114	-	-
ZtSSP79	*Mycgr3G103393*	284	CAP_euk	-	+
ZtSSP80	*Mycgr3G102849*	285	GH64-TLP-SF	-	+
ZtSSP81	*Mycgr3G76021*	279	ZnMc	-	-
ZtSSP82	*Mycgr3G48129*	96	Hydrophobin_2	+	+
ZtSSP83	*Mycgr3G43394*	232	Cutinase	-	+
ZtSSP84	*Mycgr3G98580*	270	CFEM	-	+
ZtSSP85	*Mycgr3G111221*	232	PRK06347	-	+
ZtSSP86	*Mycgr3G110386*	194	LysM	-	+
ZtSSP87	*Mycgr3G76589*	240	Glyco_hydro_45	+	+

Table S4. List of Primer used in this study.

Primer	Primer Sequence (5' - 3')	Purpose	Source
ΔSP105265 F	GGGGACAAGTTTGTACAAAAAAGCAGGCTTCATGCAAAACTACTCC GTCGGC	Cloning	This study
105265 F	GGGGACAAGTTTGTACAAAAAAGCAGGCTTCATGCGTTCCTTCATC GTTGT	Cloning	This study
105265R	GGGGACCACTTTGTACAAGAAAGCTGGGTGCAGAGCCAAGCCGA AAATGG	Cloning	This study
UBQ F	GGGG ACA AGT TTG TAC AAA AAA GCA GGC TTC ATGGCTGCTCATCCTGTTGAAT	Cloning	This study
UBQ R	GGGG AC CAC TTT GTA CAA GAA AGC TGG GTG TTACAGCTGTGATTCGATGTCATTTC	Cloning	This study
$UBQ^{126-219}$ F	GGGGACAAGTTTGTACAAAAAAGCAGGCTTCATGCCGCATTCTCC ATTATTTGA	Cloning	This study
$UBQ^{126-219}$ R	GGGGACCACTTTGTACAAGAAAGCTGGGTGTTAGTGATCGGCATA AGCTTG	Cloning	This study
105265F	TCACCGACTACACCGAGATG	qRT-PCR	This study
105265R	CGTGGAGGAGGAAGGAGAAG	qRT-PCR	This study
Mg Tub F	ATCTACCGCGGAAAGGTGTCCA	qRT-PCR	Rudd *et al.,* 2015
Mg Tub R	TGGTCGCCGACACGCTTAAAGAG	qRT-PCR	Rudd *et al.,* 2015
35s F (CD3-687)	CCTTCGCAAGACCCTTCCTC	Cloning	This study
OCS R (CD3-687)	GACATGTTGTCGCAAAATTCGCC	Cloning	This study
UBQV1F	CGATTAATTAACTAATGAGCTTTCGTGAAGATCT	VIGS	This study
UBQV1R	CGACCCGGGGGGGCATAGTGCGTTTATC	VIGS	This study
UBQV2F	CGATTAATTAACAACCGCCCAACAACTG	VIGS	This study
UBQV2R	CGACCCGGGAGTCAAGGGCCATCCTGAA	VIGS	This study
QPCR F1	GCGGCCATTTGTGTTCTGTC	VIGS	This study
QPCR R1	GCAACACAACCTACCGTATA	VIGS	This study
TaCDC48F	AAATACGCCATCAGGGAGAACATCGAG	qRT-PCR HK VIGS	Lee *et al.,*2014
TaCDC48R	CTCGCTGCCGAAACCACGAGAC	qRT-PCR HK VIGS	Lee *et al.,*2014
TaeIF4E F	GATTGAGCCAAAATGGGAAGAC	qRT-PCR HK VIGS	Lee *et al.,*2014
TaeIF4E R	GCCAGCAAAGTATGCAACCA	qRT-PCR HK VIGS	Lee *et al.,*2014
38105 (Zt-6) F	GGGGACAAGTTTGTACAAAAAAGCAGGCTTCATGCGTTTCTCCCTC CTCTC	Cloning	Kettles et al., 2017
38106 (Zt-6) R	GGGGACCACTTTGTACAAGAAAGCTGGGTGACTAGCTTGTGCCCG AACAC	Cloning	Kettles et al., 2017
RcSSP2 F	GGGGACAAGTTTGTACAAAAAAGCAGGCTTCATGCAGAACACAAC TGTCGAG	Cloning	This study
RcSSP2 R	GGGGACCACTTTGTACAAGAAAGCTGGGTGCTACAACGCCAGACC GAAGAT	Cloning	This study
HvUBQF	GGGG ACA AGT TTG TAC AAA AAA GCA GGC TTCATGGCTGCTCATCCTGTTGAAT	Cloning	This study
HvUBQR	GGGG AC CAC TTT GTA CAA GAA AGC TGG GTGTTACAGCTGCGATTCGATGTCATTTC	Cloning	This study
111505 (Zt-10)F	GGGGACAAGTTTGTACAAAAAAGCAGGCTTCCTCAGACGACCCAG TCTGCAC	Cloning	This study
111505 (Zt-10)R	GGGGACCACTTTGTACAAGAAAGCTGGGTGCTAATAGGCCGCAGA GTATC	Cloning	This study

Table S6. List of wheat proteins identified as a potential interactor with *Z. tritici* candidate ZtSSP2. Sequences obtained from positive clones in yeast 2 hybrid assay was blasted against Ensemble wheat (TGACv1) dataset and corresponding full-length sequences were obtained.

**BLAST against *T.aestivum* TGACv1**	**Best BLASTP match**	**AA Size**
TRIAE_CS42_1DS_TGACv1_082290_AA0264360.1	E3 ubiquitin-protein ligase At1g12760-like (LOC109745851)	420
TRIAE_CS42_7DL_TGACv1_604456_AA1998280.4	Endoplasmin homolog (LOC109732828)	634
TRIAE_CS42_7AL_TGACv1_556087_AA1755000.3		
TRIAE_CS42_7BL_TGACv1_578233_AA1891510.4		
TRIAE_CS42_1BL_TGACv1_030593_AA0095040.1	Heavymetal-associated isoprenylated plant protein 39-like (LOC109766401)	224
TRIAE_CS42_1AL_TGACv1_000743_AA0018220.1		
TRIAE_CS42_1BL_TGACv1_030333_AA0087160.1	Endo-1,3;1,4-beta-D-glucanase-like (LOC109778325)	240
TRIAE_CS42_1DL_TGACv1_061296_AA0191610.1		
TRIAE_CS42_5DL_TGACv1_435835_AA1455370.1	Ankyrin repeat domain-containing protein 2A-like (LOC109746370)	398
TRIAE_CS42_5BL_TGACv1_406247_AA1342930.1		
